# Influence of genomic structural variations and nutritional conditions on the emergence of quorum sensing-dependent gene regulation defects in *Burkholderia glumae*

**DOI:** 10.3389/fmicb.2022.950600

**Published:** 2022-07-15

**Authors:** Minhee Kang, Jae Yun Lim, Jinwoo Kim, Ingyu Hwang, Eunhye Goo

**Affiliations:** ^1^Department of Agricultural Biotechnology, Seoul National University, Seoul, South Korea; ^2^School of Systems Biomedical Science, Soongsil University, Seoul, South Korea; ^3^Department of Plant Medicine and Institute of Agriculture and Life Sciences, Gyeongsang National University, Jinju, South Korea; ^4^Research Institute of Agriculture and Life Sciences, Seoul National University, Seoul, South Korea

**Keywords:** *Burkholderia glumae*, genome variation, *qsmR* mutant, adaptation, nutrient stress

## Abstract

Bacteria often change their genetic and physiological traits to survive in harsh environments. To determine whether, in various strains of *Burkholderia glumae*, genomic diversity is associated with the ability to adapt to ever-changing environments, whole genomes of 44 isolates from different hosts and regions were analyzed. Whole-genome phylogenetic analysis of the 44 isolates revealed six clusters and two divisions. While all isolates possessed chromosomes 1 and 2, strains BGR80S and BGR81S had one chromosome resulting from the merging of the two chromosomes. Upon comparison of genomic structures to the prototype BGR1, inversions, deletions, and rearrangements were found within or between chromosomes 1 and/or 2 in the other isolates. When three isolates—BGR80S, BGR15S, and BGR21S, representing clusters III, IV, and VI, respectively—were grown in Luria-Bertani medium, spontaneous null mutations were identified in *qsmR* encoding a quorum-sensing master regulator. Six days after subculture, *qsmR* mutants were found at detectable frequencies in BGR15S and BGR21S, and reached approximately 40% at 8 days after subculture. However, the *qsmR* mutants appeared 2 days after subculture in BGR80S and dominated the population, reaching almost 80%. No *qsmR* mutant was detected at detectable frequency in BGR1 or BGR13S. The spontaneous *qsmR* mutants outcompeted their parental strains in the co-culture. Daily addition of glucose or casamino acids to the batch cultures of BGR80S delayed emergence of *qsmR* mutants and significantly reduced their incidence. These results indicate that spontaneous *qsmR* mutations are correlated with genomic structures and nutritional conditions.

## Introduction

In natural microbial populations, physiologically or genetically distinct variants often exist, having evolved by adapting to the environmental conditions of their natural habitat (Achtman and Wagner, 2008). Closed batch cultures provide an excellent, easy to use laboratory system for studying adaptive variants to nutrient stress, because bacterial cells can be cultured for several days without the addition of nutrients (Ratib et al., 2021). The relative fitness benefits of these variants, and their strategies for adapting to various stresses, have been studied in different microorganisms (Helsen et al., 2020; Aggeli et al., 2021).

*Burkholderia glumae* has a wide range of hosts, including rice, tomato, pepper, and potato, causing rice panicle blight and bacterial wilt (Jeong et al., 2003). Although *B*. *glumae* is known as a plant pathogen, the clinical isolate *B*. *glumae* strain AU6208 has been reported from patients with chronic infections (Weinberg et al., 2007). The sequenced genomes of plant-associated *B*. *glumae* strains, including 336gr-1, LMG2196, and PG1, and the clinical isolate AU6208, were compared with that of *B*. *glumae* BGR1 isolated from an infected rice plant in South Korea (Francis et al., 2013; Lee et al., 2016; Hussain et al., 2020). These comparative studies revealed that while the isolates share common characteristics, such as toxoflavin biosynthesis genes, unique genomic regions and large-scale genome rearrangement and inversion were observed in each strain (Francis et al., 2013; Lee et al., 2016; Hussain et al., 2020). However, whether the genotypically distinct isolates of *B*. *glumae* have distinctive physiological traits and evolutionary routes for environmental adaptation is unknown.

*B*. *glumae* has a single LuxIR-type TofIR quorum sensing (QS) system that regulates the biosynthesis of toxoflavin, a key virulence factor, and activates the expression of the IclR-type transcription regulator *qsmR* (Kim et al., 2004, 2007). QsmR regulates the expression of dozens of genes, including flagella and oxalate biosynthesis genes, in a TofIR-dependent manner (Kim et al., 2007; Goo et al., 2012). When *B*. *glumae* is cultured in Luria-Bertani (LB) medium, ammonia accumulates due to deamination using amino acids as a carbon source (Goo et al., 2012). To neutralize the alkaline toxicity mediated by ammonia, oxalate is biosynthesized in a QsmR-dependent manner (Goo et al., 2012). These results allowed us to investigate how QS-dependent physiological traits differ among *B*. *glumae* isolates originating from different hosts and regions.

In this study, we comparatively analyzed the genomes of 36 Korean isolates and 8 previously known isolates of *B*. *glumae*, and investigated the physiological traits of the Korean isolates. No significant differences in QS-dependent physiological traits, such as virulence, biosynthesis of toxoflavin, oxalate, and QS signals, were found among the 36 isolates. However, depending on the genome structure, *qsmR* mutants appeared at various frequencies in closed batch culture of isolates after the stationary phase, and daily supplementation of the batch culture with carbon sources reduced the frequency of *qsmR* mutant appearance. These results provide insight into how *B*. *glumae* evolves in harsh environments, as well as how this adaptive evolution leads to QS-defective phenotypes.

## Materials and methods

### Bacterial strains and growth conditions

The bacterial isolates used in this study are listed in Table 1. *B. glumae* isolates were grown in LB broth [0.1% tryptone, 0.5% yeast extract, and 0.5% NaCl (w/v); USB, Cleveland, OH, United States] or in LB broth buffered with 100 mM HEPES (pH 7.0) at 37^°^C. When necessary, the LB broth was daily supplemented with 2% glucose and 5% casamino acid for growth experiments.

**TABLE 1:** *B. glumae* isolates used in this study.

Isolate	Genome size (bp)		No. of plasmids	GC%	Geographical origin^a^	Accession number
	Chr. 1	Chr. 2				
**BGR1**	3,911,671	2,826,231	4	68		SAMN02603166
BGR33S	3,791,140	2,812,129	2	68	Haenam-gun, Jeollanam-do	SAMN29352428
BGR39S	3,726,678	2,828,244	4	68	Chungju-si, Chungcheongbuk-do	SAMN29352429
BGR49S	3,912,005	2,828,244	4	68		SAMN29352430
BGR57S	3,912,005	2,828,244	4	68	Changnyeong-gun, Gyeongsangnam-do	SAMN29352431
BGR59S	3,912,005	2,828,244	4	68	Goryeong-gun, Gyeongsangbuk-do	SAMN29352432
BGR68S	3,912,005	2,828,244	4	68	Bonghwa-gun, Gyeongsangbuk-do	SAMN29352433
BGR73S	3,912,005	2,828,244	4	68	Hapcheon-gun, Gyeongsangnam-do	SAMN29352434
BGR82S	3,912,005	2,828,244	4	68	Muju-gun, Jeollabuk-do	SAMN29352435
BGR85S	3,738,765	2,820,186	3	68	Naju-si, Jeollanam-do	SAMN29352436
BGR86S	3,625,957	2,852,417	5	68	Suwon-si, Gyeonggi-do	SAMN29352437
R5	3,673,124	2,846,341	3	68	Sacheon-si, Gyeongsangnam-do	SAMN29352438
SL-947S	3,738,765	2,820,186	3	68		SAMN29352439
ATCC33617*	3,659,771	2,820,414	2	68	Ehime, Japan	SAMN03012983
FDAARGOS_949*	3,659,799	2,820,518	2	68		SAMN13450479
FDAARGOS_921*	3,659,785	2,820,468	2	68		SAMN13450451
TW34RS	3,912,005	2,828,244	4	68	Gimje-si, Jeollabuk-do (Tomato)	SAMN29352440
PERW1RS	3,912,004	2,828,243	4	68	Buyeo-gun, Chungcheongnam-do (Perilla)	SAMN29352441
PW30RS	3,912,005	2,872,561	4	68	Hadong-gun, Gyeongsangnam-do (Pepper)	SAMN29352442
EW10RS	3,912,005	2,828,244	4	68	Seocheon-gun, Chungcheongnam-do (Eggplant)	SAMN29352443
SW2RS	3,912,006	2,828,245	4	68	Jecheon-si, Chungcheongbuk-do (Sunflower)	SAMN29352444
POW17RS	3,912,004	2,828,243	4	68	Yeongam-gun, Jeollanam-do (Potato)	SAMN29352445
SEW1RS	3,912,006	2,828,245	4	68	Buan-gun, Jeollabuk-do (Sesame)	SAMN29352446
**BGR15S**	3,742,794	2,880,619	4	68	Cheongju-si, Chungcheongbuk-do	SAMN10390411
BGR19S	3,690,419	2,852,417	3	68	Hadong-gun, Gyeongsangnam-do	SAMN29352447
BGR22S	3,827,400	2,836,302	4	68	Buan-gun, Jeollabuk-do	SAMN29352448
BGR28S	3,779,053	2,796,013	4	68	Gyeongsan-si, Gyeongsangbuk-do	SAMN29352449
BGR35S	3,702,773	2,834,421	4	68	Miryang-si, Gyeongsangnam-do	SAMN29352450
BGR76S	3,593,726	2,880,619	4	68	Namwon-si, Jeollabuk-do	SAMN29352451
YH8	3,764,216	2,908,152	3	68	Goseong-gun, Gangwon-do (diseased broken rice)	SAMN29352452
GX*	3,712,850	2,750,046	3	68	GuangXi, China	SAMN13005961
**BGR21S**	3,815,313	2,787,956	3	68	Changwon-si, Gyeongsangnam-do	SAMN10390410
BGR79S	3,734,736	2,783,927	4	68	Jangsu-gun, Jeollabuk-do	SAMN29352453
HN2*	3,532,897	2,745,532	1	68	Hunan, China	SAMN13005960
257sh-1*	3,537,953	2,763,738	2	68	Louisiana, United States	SAMN10913710
BGR48S	3,625,957	2,735,580	4	68	Suwon-si, Gyeonggi-do	SAMN29352454
R2	3,651,865	2,728,074	3	68	Haman-gun, Gyeonsangnam-do	SAMN29352455
R93	3,644,544	2,808,990	2	68	Taean-gun, Chungcheongnam-do	SAMN29352456
AU6208*	3,629,551	2,779,201	1	68		SAMN13567886
HN1*	3,611,155	2,869,204	3	68		SAMN14694445
SL-2395S	3,775,023	2,840,330	2	68		SAMN29352457
**BGR13S**	4,105,390	2,248,089	3	68	Gangjin-gun, Jeollanam-do	SAMN10390412
**BGR80S**	6,450,182		4	68	Suwon-si, Gyeonggi-do	SAMN10390413
BGR81S	6,490,471		4	68	Icheon-si, Gyeonggi-do	SAMN29352458

^a^Hosts from which the strain was isolated are in parentheses; all unmarked cases were isolated from diseased rice panicles. The isolates of bold highlighting are the representatives of each group.

*Isolates used only for comparative genome analysis.

### Genome sequencing and assembly

Thirty-three *B*. *glumae* isolates originating from South Korea were sequenced using PacBio RS-II single molecule real-time (SMRT) technology. The SMRTbell library was subjected to size selection for quality control using the BluePippin system. The genomic DNA of strains R2, R5, and R93 was extracted using a previously published method (Sambrook et al., 1989) and sequenced using PacBio RSII and the Illumina platform (Illumina, San Diego, CA, United States). The datasets were assembled using FALCON-integrate (v2.1.4).^1^ PacBio reads were assembled, once with HGAP v. 3 (Chin et al., 2013). HGAP3 was run on SMRT analysis v.2.3.0 and protocol RS HGAP assembly.3 with data of all four SMRT cells (default parameters, except estimated genome size: 7.35 Mb). Following draft assembly, contigs were analyzed for terminal overlaps with Circlator. Overlapping regions were trimmed and the resulting circular chromosomes and plasmids were reoriented and cut such that the origin of replication was at the start: chromosome 1 was started at the 100 bp upstream of *dnaA* gene, and chromosome 2 and plasmids were at the 100 bp upstream of *parA* gene. Circularized and reoriented was polished with Quiver (Chin et al., 2013) with SMRT cells again. Complete genome sequences of eight strains (ATCC33617, FDAARGOS_949, FDAARGOS_921, GX, HN1, NH2, 257sh-1, AU6208) were downloaded from NCBI (20 April 2021) for comparative analysis.

### Phylogenetic analysis

Phylogenetic analysis was carried out based on the chromosomal sequence homology. Plasmid sequences were not used for this analysis. Average nucleotide identity based on BLAST (ANIb) values was calculated using the JSpecies Standalone JAVA program (V1.2.1). A file created in Newick tree format by the hclust (hierarchical clustering) method in R software (R Development Core Team, Vienna, Austria), drawn using MegaX, was used to generate a phylogenetic tree.

### Comparative genomic analyses

Chromosome sequences were used for synteny and single nucleotide polymorphism (SNP) analyses. Synteny and SNP data were analyzed using the dnadiff program of the MUMmer package v4.0. A synteny map was drawn using Python script based on the MUMmer analysis. All 44 *B. glumae* genome sequences were employed for a pan- and core-genome analyses, using the pan-genomes analysis pipeline (PGAP) v1.1 (Zhao et al., 2012) and the Gene Family method with 75% identity and 50% coverage options.

### Kanamycin resistance

After dilution of *B. glumae* isolates cultured in LB broth, spotting of the diluted culture solution on agar medium containing kanamycin was performed to confirm whether the bacteria could grow in the medium with antibiotics. In the antibiotics resistance assay, kanamycin was used at a concentration of 50 mg/mL. The kanamycin resistance gene was confirmed by an NCBI DB search for the nucleotide sequence of the long insertion region from the BLAST result for isolates with kanamycin resistance.

### Virulence assay

The bacterial cells [1 × 10^8^ colony forming units (CFU)/mL] were inoculated into Milyang-23 rice plants to assess virulence. The plants were kept in a growth chamber for 7 days at 30°C with a 16-h/8-h light/dark regime for virulence testing. The disease index was calculated using ImageJ 1.53 a software (NIH, Bethesda, MD, United States) by quantifying pixels in the diseased area. The relative disease index was calculated by comparison with wild type values (disease index = disease pixels/disease pixels from the wild type). The virulence assay was repeated three times with three independent replicates.

### Toxoflavin and quorum sensing signal detection

A toxoflavin and QS signal detection were performed as described previously (Kim et al., 2004).

### Bacterial population and extracellular pH assay

Bacterial population and extracellular pH were monitored as described previously (Goo and Hwang, 2021).

### Measurements of ammonia and oxalate levels

The levels of ammonia and oxalate were measured as described previously (Goo et al., 2012).

### Identification of the mutations in spontaneous null mutants

Chromosomal DNA was isolated from 21 spontaneous mutants cultured in LB broth as described previously (Sambrook et al., 1989). Based on our previous studies and the phenotypes of spontaneous null mutants, the *tofIR*, *qsmR*, and *obcAB* genes were selected as gene mutation candidates. We amplified the *tofIR*, *qsmR*, and *obcAB* gene regions of 21 mutants using the tofI-F, tofR-R, qsmR-F, qsmR-R, obcA-F, and obcb-R primers (Supplementary Table 1). No mutations were identified in the *tofIR* or *obcAB* gene regions. Mutations in the *qsmR* gene representing four different mutation types, i.e., insertion, small deletion, medium deletion, and large deletion, were detected. We confirmed the position of 29 insertion mutants, 2 small deletion mutants, 3 medium deletion mutants, and 1 large deletion mutant by direct sequencing.

## Results

### Identification and genome sequencing of *Burkholderia glumae* isolates

*B*. *glumae* isolates were collected from all provinces of South Korea, including Gyeonggi-do, Gyeongsang-do, Jeolla-do, Chungcheong-do, and Gangwon-do (Table 1). Among 36 *B*. *glumae* isolates, 28 samples were isolated from diseased rice panicles, and YH8 was isolated from diseased broken rice. The other seven samples—TW34RS, PERW1RS, PW30RS, EW10RS, SW2RS, POW17RS, and SEW1RS—were from Solanaceous crops such as tomato, perilla, pepper, eggplant, sunflower, potato, and sesame (Table 1). Among the 36 Korean *B*. *glumae* isolates, the complete genome sequence of strain BGR1 had been determined previously (Lim et al., 2009). The remaining 35 isolates shared more than 99% identity with *B*. *glumae* based on 16S rRNA analysis, and the complete genome sequences of these isolates were determined. The genomes of the 35 isolates ranged in size from 6.5 to 7.3 Mbp, with a G + C content of 68% (Table 1). Unlike the other isolates that have two chromosomes, BGR80S and BGR81S have one large chromosome (Table 1). The number of plasmids in the *B*. *glumae* isolates varied from one to five (Table 1).

### Comparative genome analysis of *Burkholderia glumae* isolates

**FIGURE 1:**
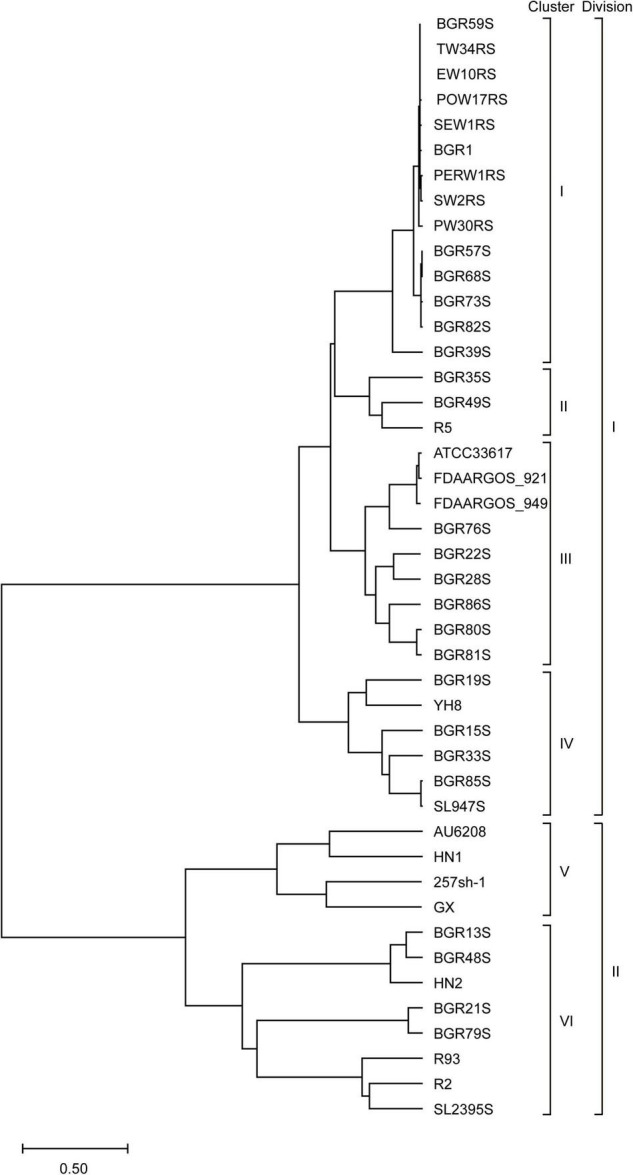
Phylogenetic analysis of *B. glumae* isolates based on the homology of two chromosome sequences. According to their sequence homology, 44 *B. glumae* genomes were classified into two divisions and six clusters.

**FIGURE 2:**
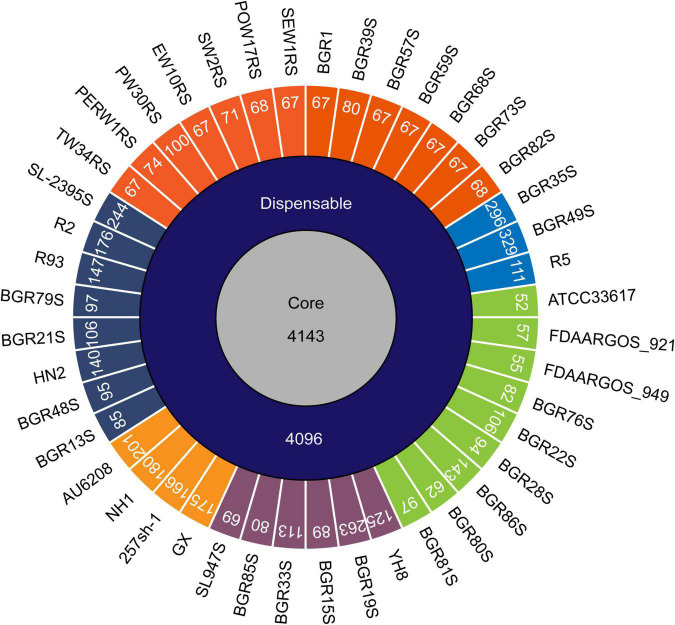
Pan-genome analysis of *B. glumae* isolates. The numbers inside the circular diagram indicate the numbers of core, dispensable, and unique genes. The unique genes of each isolate are colored according to their phylogenetic classification; cluster I, orange; cluster II, blue; cluster III, green; cluster IV, purple; cluster V, light-orange; cluster VI, navy.

To determine whether the *B. glumae* strains from different hosts and regions have genomic diversity, the genomes of 44 isolates were comparatively analyzed. Based on a phylogenetic analysis comparing chromosome sequence homology, the 44 isolates were classified into six clusters and two divisions; division I contained clusters I–IV, while clusters V and VI were in division II (Figure 1). Strain BGR1 and the isolates from Solanaceous crops were in cluster I; BGR35S, BGR49S, and R5 were in cluster II; BGR80S and BGR81S were in cluster III; YH8 isolated from diseased broken rice and BGR15S were in cluster IV; HN1, 257sh-1, GX, and the clinical isolate AU6208 were in cluster V; BGR13S and BGR21S were in cluster VI (Figure 1). Pan-genome analysis via PGAP identified 13,298 orthologs in all isolates (Figure 2). Among them, 4,143 orthologs were highly conserved, constituting the core genome (Figure 2). Another 4,096 orthologs were identified as dispensable, while the individual genomes contained 52–329 strain-specific genes (Figure 2).

### Genomic structural variation across *Burkholderia glumae* isolates

**FIGURE 3:**
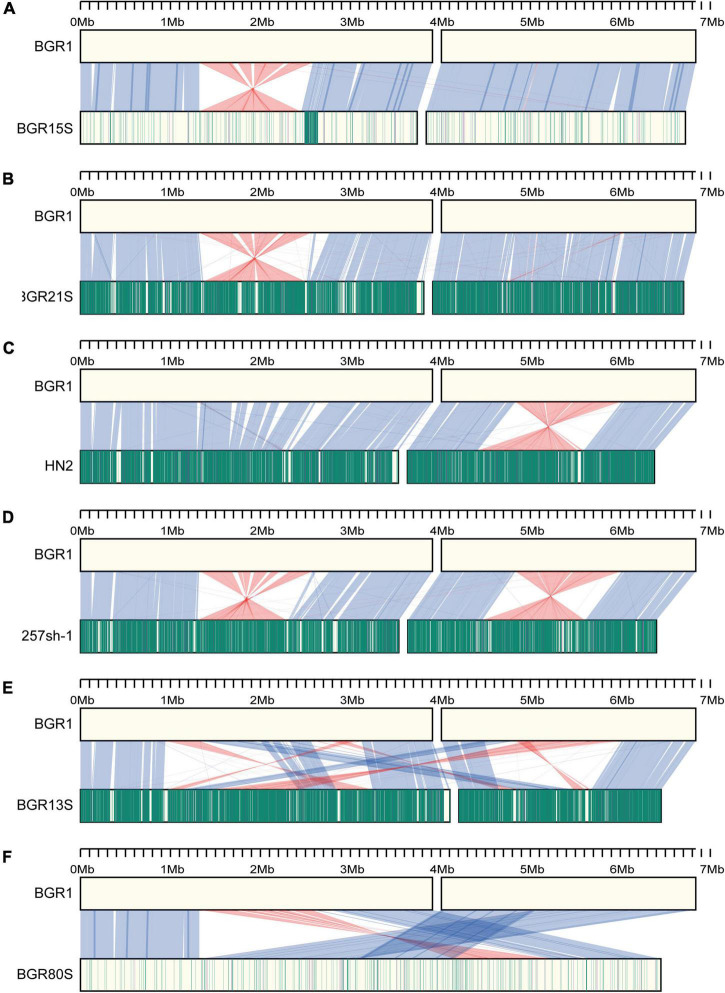
Synteny analysis of *B. glumae* isolates. **(A–F)** Genome structures of each isolate are compared to BGR1. The upper part is the BGR1 genome; the lower part is the genomes of other *B. glumae* isolates; **(A)** BGR15S, **(B)** BGR21S, **(C)** HN2, **(D)** 257sh-1, **(E)** BGR13S, and **(F)** BGR80S. The blue lines between genomes of two different isolates show the regions corresponding to each other; the red lines show areas where inversion occurred. The vertical green lines indicate the locations of SNPs compared to BGR1; the vertical purple lines indicate INDELs. The darker color, the more SNPs or INDELs occurred.

Synteny analysis was performed on the assembled sequences to improve the gene annotation of the 35 newly sequenced isolates, and to study the genomic evolution in all 44 isolates. Using structural homologies of the genome, the 44 isolates were divided into seven groups, represented by isolates BGR1, BGR15S, BGR21S, HN2, 257sh-1, BGR13S, and BGR80S. The BGR1 group consisted of isolates with a similar genome structure to the prototype BGR1 (Supplementary Figure 1). The isolates of the BGR15S group had one inversion on chromosome 1 g (except isolate GX, which had two inversions), as well as several deletions in the genome (Figure 3A and Supplementary Figure 1). In the BGR21S group, BGR21S and BGR79S also had one inversion on chromosome 1 g, but there was a sequence discrepancy with BGR1, with sequence homologies of 99.18% (BGR21S) and 99.21% (BGR79S) (Figure 3B and Supplementary Figure 1). The HN2 group isolates had an inversion on chromosome 2 g, as well as several deletions in the genome (Figure 3C). The 257sh-1 group isolates each had inversions on chromosomes 1 and 2 g, but the inversion positions were all different; strain R93 had one inversion on chromosome 1 g and three on chromosome 2 g (Figure 3D and Supplementary Figure 1). Compared to BGR1, the BGR13S group isolates had rearrangements at 24 positions between chromosomes 1 and 2 g (Figure 3E and Supplementary Figure 1). The BGR80S group isolates possessed two chromosomes that were merged into one (Figure 3E and Supplementary Figure 1).

### Merged two chromosomes in the BGR80S group genome

**FIGURE 4:**
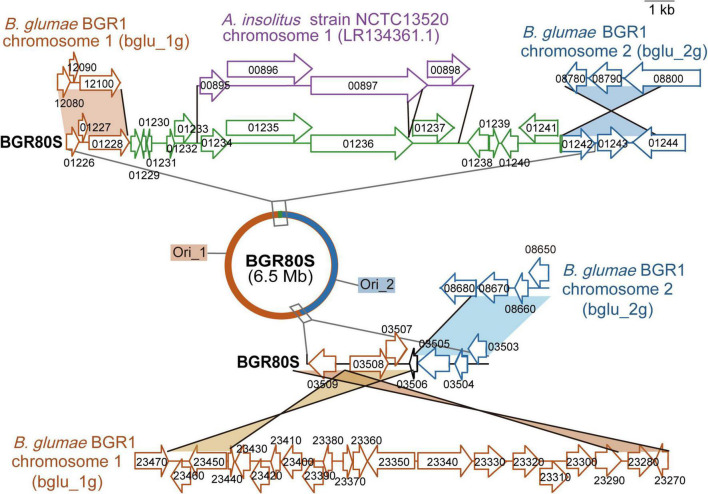
Genetic map of the fusion junctions of two chromosomes in *B. glumae* BGR80S. The red half-circle is the region corresponding to prototype BGR1 chromosome 1 (bglu_1g); the blue half-circle is the area corresponding to BGR1 chromosome 2 (bglu_2g). On the two chromosomes of BGR1, the genes bglu_1g 12100 and bglu_2g 08800, and bglu_1g 23470 and bglu_2g 08680, merged to form the single chromosome of BGR80S. There were two replication origins in the single chromosome of BGR80S. A DNA fragment of *A. insolitus* strain NCTC13520 chromosome 1 was inserted between bglu_1g01228 and bglu_2g01242 in the BGR80S genome. The DNA fragment in purple is the original sequence from *A*. *insolitus* strain NCTC13520; the DNA fragment in green with some deletions was inserted into *B*. *glumae* BGR80S.

To determine how the single large chromosomes of BGR80S and BGR81S were generated, we investigated the genetic exchanges that occurred at junctions between chromosomes 1 and 2 g. At one location, transposase (bglu_1 g12100) and fimbrial biogenesis outer membrane usher protein (bglu_2 g08800) genes were combined (Figure 4). In addition, transposases, integrases, and a 9,533-bp DNA fragment with 89% sequence identity to *Achromobacter insolitus* strain NCTC13520 were inserted between the two genes (Figure 4). In another location, the L-seryl-tRNA selenium transferase gene (bglu_2 g08680) and transposase IS66 (bglu_1 g23470) were merged (Figure 4). In BGR80S, a 39,497-bp sequence present in the BGR81S genome (base range: 5,377,980–5,417,476) was also deleted (Table 1). The *ori1* and *ori2* origins of replications were identified in BGR80S and BGR81S by homology to the chromosome 1 and 2g origins of prototype BGR1 (Figure 4).

### Kanamycin resistance

**FIGURE 5:**
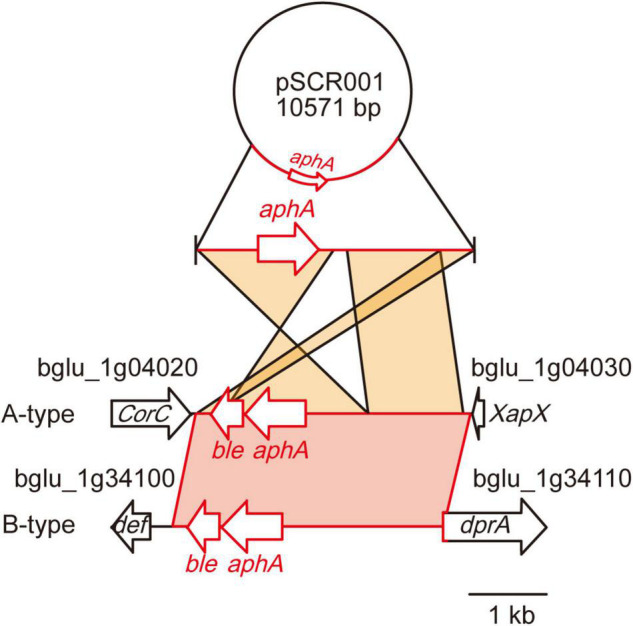
Genetic map of the insertion of the kanamycin resistance gene. A DNA fragment containing a kanamycin resistance gene (*aphA*) derived from plasmid pSCR001 was inserted in two different types of regions: A-type, between bglu_1g04020 and 1g04030 in BGR57S, BGR68S, BGR73S, BGR82S; and B-type, between bglu_1g34100 and 1g34110 in BGR59S. The DNA fragments inserted in *B. glumae* isolates showed 99% identity to pSCR001.

To evaluate whether the 36 *B*. *glumae* isolates had acquired antibiotic resistance genes, we tested for antibiotic resistance. Five isolates from cluster I—BGR57S, BGR59S, BGR68S, BGR73S, and BGR82S—exhibited resistance to kanamycin and contained the kanamycin resistance gene aminoglycoside 3′-phosphotransferase (*aphA*) in their genomes (Figure 5 and Supplementary Figure 2). The foreign DNA carrying the *aphA* gene was inserted between the bglu_1 g04020 gene and bglu_1 g04030 genes in BGR57S, BGR68S, BGR73S, and BGR82S (Figure 5). In BGR59S the gene was inserted into the bglu_1g34110 gene (Figure 5). BLAST analysis revealed that the inserted DNA sequence matched the plasmid pSCR001 (accession no. DQ059989) with the widest query coverage (Figure 5).

### Emergence of quorum sensing-dependent gene regulation defects

**FIGURE 6:**
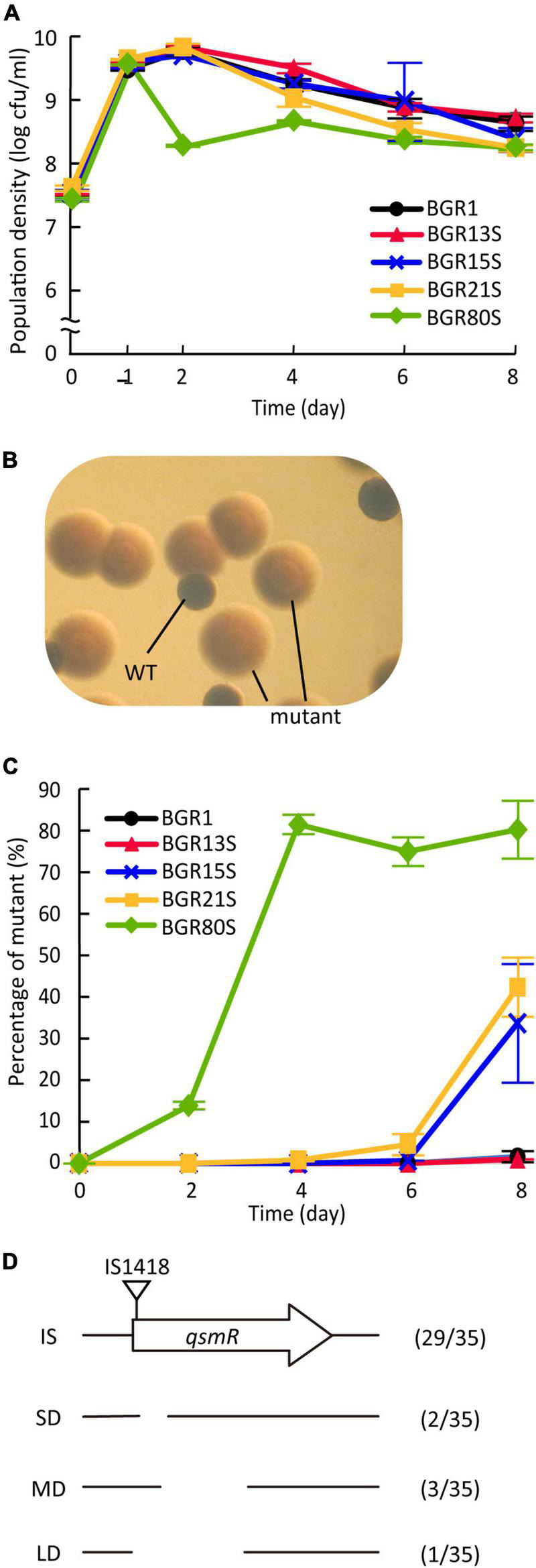
Cell viability and appearance of quorum sensing-dependent gene regulation defects from *B. glumae* isolates. **(A)** Viable cell numbers were monitored at 1, 2, 4, 6, and 8 days after subculture and colony-forming units (CFUs) were counted. Data represent the mean ± standard error (SE) of triplicates. **(B)** Morphologically distinct colonies compared to the wild type colonies were observed in BGR80S culture under a dissecting microscope at 30 × magnification. **(C)** The percentage of mutants was monitored at 2-day intervals for 8 days, and calculated by dividing the CFUs of the mutants by the total CFUs. Data represent the mean ± *SE* of triplicates. **(D)** Characterization of *qsmR* mutations in mutants emerging from BGR80S, BGR15S, and BGR21S. Among the 35 mutants, 29 had an insertion of IS1418 at the 5’ end of the *qsmR* gene, and 2, 3, and 1 had 112-, 361-, and 468-bp internal deletions, respectively.

Since the genomic diversity of *B. glumae* isolates did not affect their virulence, or the biosynthesis of QS signals and toxoflavin (Supplementary Figures 3, 4), we explored whether the genomic diversity of *B. glumae* isolates was associated with their ability to adapt to changing environments. We monitored the populations of BGR1, BGR13S, BGR15S, BGR21S, and BGR80S as representative isolates of each genome group during an 8-day incubation in LB medium, without supplementation with additional nutrients. The population density of BGR80S decreased 1 day after subculture, and was then maintained at about 3.1 × 10^8^ CFU/mL (Figure 6A). The other three isolates showed the same growth pattern as BGR1 (Figure 6A). All five isolates exhibited the same patterns in extracellular pH (Supplementary Figure 5). However, the populations of BGR80S, BGR15S, and BGR21S were not homogeneous, containing morphologically distinct mutant colonies (Figure 6B). The mutant appeared 2 days after subculture in the BGR80S and soon accounted for ∼80% of the population (Figure 6C). In BGR15S and BGR21S, the mutant was detected 6 days after subculture and reached ∼40% of the population (Figure 6C). No mutant was observed at detectable frequency in BGR1 or BGR13S (Figure 6C). The mutants did not survive the stationary phase after being isolated due to the toxic alkaline extracellular pH resulting from the failure to activate oxalate biosynthesis in a QS-dependent manner, as observed previously (Supplementary Figure 6). To characterize the spontaneously occurring mutant, we sequenced the *tofIR*, *qsmR*, and *obcAB* gene regions associated with its phenotype. All 35 isolates from three experiments in which mutants accumulated had *qsmR* mutations (Figure 6D). The *qsmR* mutations arising in BGR80S, BGR15S, and BGR21S included IS element insertions, small deletions, medium deletions, and large deletion in the *qsmR* gene (Figure 6D).

### The mutant has a fitness benefit over the wild type

**FIGURE 7:**
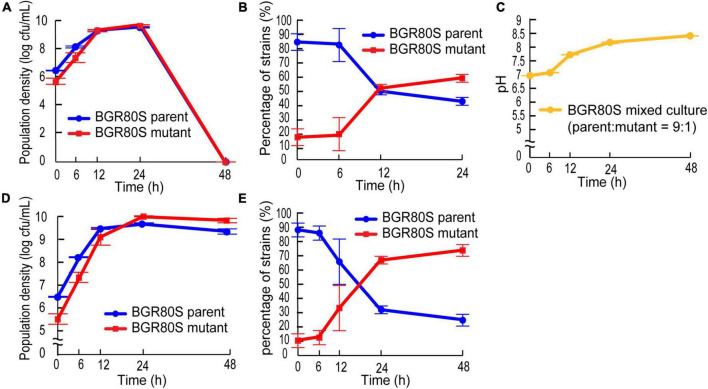
Competition between the parental strain BGR80S and the mutant in fresh LB or LB supplemented with 100 mM HEPES (pH 7.0). **(A)** Viable cell numbers of the parent and mutant in LB medium were monitored by counting CFUs at 0, 6, 12, 24, and 48 h after subculture with an initial mutant frequency of ∼10%. **(B)** The proportions of parent and mutant in LB medium were calculated by dividing their CFUs by the total CFUs. If no viable cells were observed 48 h after subculture in LB medium, the percentage of strains was not counted. **(C)** External pH fluctuation during this experiment. **(D)** Viable cell numbers of the parent and mutant in LB supplemented with 100 mM HEPES (pH 7.0) were determined by counting CFUs at 0, 6, 12, 24, and 48 h after subculture with an initial mutant frequency of ∼10%. **(E)** The proportion of parent and mutant in LB supplemented with 100 mM HEPES (pH 7.0) were calculated by dividing their CFUs by the total CFUs. All data represent the mean ± SE of triplicates.

To determine whether the mutation in QS-dependent gene regulation confers a fitness advantage during batch culture incubation, we monitored the population density and pH in mixed culture. The mutant outcompeted the parent even when the initial mutant frequency was ∼10% in fresh LB medium (Figures 7A,B). The percentage of mutants increased from 16 to 58.4%, while that of BGR80S parents decreased from 84 to 41.6% during competition in the LB medium. Whole populations collapsed in LB medium because the external pH increased to ∼8.4, and viable *B*. *glumae* is not observed at a pH of 8.5 (Figures 7A,C). The addition of 100 mM HEPES buffer to LB medium prevented population collapse; the mutant then outgrew the parent in mixed culture, increasing from 10.4 to 75% of the whole population (Figures 7D,E).

### Daily nutrient supplementation reduces mutation incidence

**FIGURE 8:**
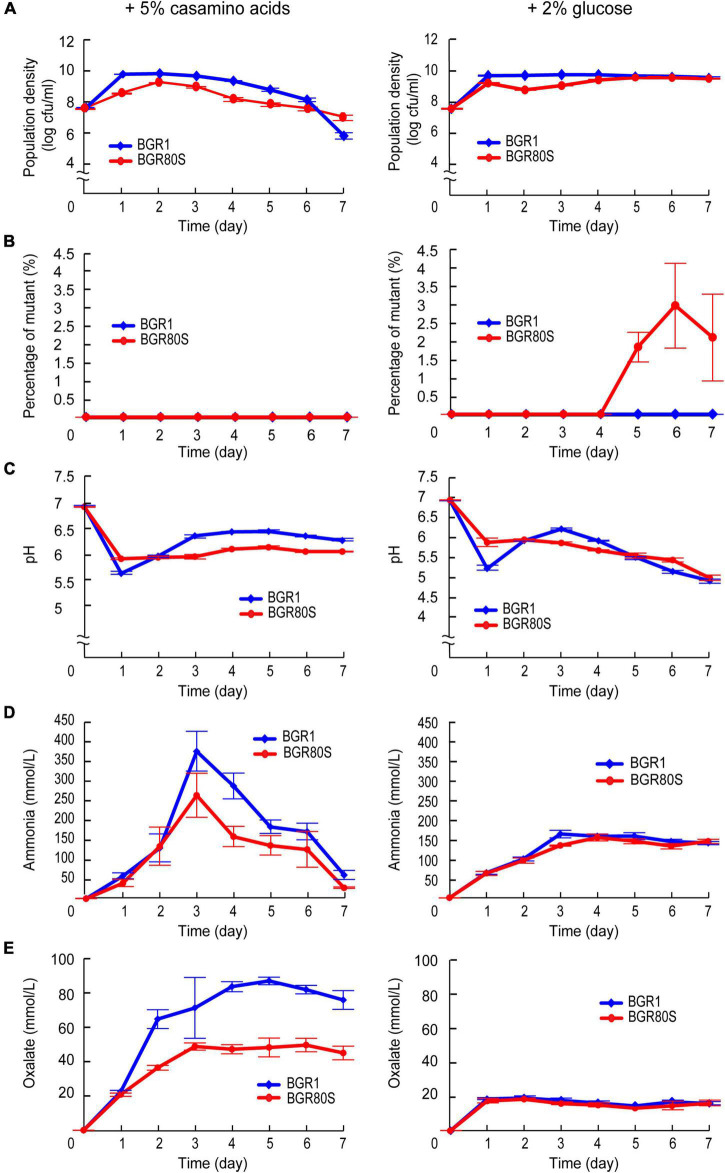
Reduced mutation incidence for BGR80S in LB supplemented with 5% casamino acids or 2% glucose. **(A)** Viable cell numbers of BGR1 and BGR80S were monitored daily for 7 days by counting CFUs during growth in LB supplemented with 5% casamino acids or 2% glucose. **(B)** The percentage of mutants was calculated by dividing the CFUs of the mutant by the total CFUs during growth in LB supplemented with 5% casamino acids or 2% glucose. **(C)** The external pH of BGR1 and BGR80S was measured for 7 days, and **(D,E)** the levels of ammonia and oxalate produced by BGR1 and BGR80S were examined for the indicative days in casamino acid- or glucose-enriched LB medium. All data represent the mean ± *SE* of triplicates.

In batch culture, nutrient stress may become constant from 1 day after subculture, inducing spontaneous mutations to adapt to these conditions. To test this hypothesis, on each day we added 5% casamino acids or 2% glucose to the batch culture in LB medium, to evaluate their influence on mutant incidence. Total populations of BGR1 and BGR80S grown in LB enriched with casamino acids reached a maximum and then gradually began to decrease 2 days after subculture, reaching ∼7.5 × 10^6^CFU/mL for BGR1 and ∼1.3 × 10^7^CFU/mL for BGR80S at 7 days (Figure 8A). BGR1 and BGR80S grown in LB enriched with glucose maintained 6.0 × 10^9^CFU/mL and 3.8 × 10^9^ (CFU/ml), respectively, for 7 days (Figure 8A). Spontaneous mutants were not observed from either strain grown in casamino acid-rich conditions (Figure 8B). However, in glucose-rich conditions, the mutant appeared 4 days after subculture in BGR80S and reached ∼2–3% of the whole population (Figure 8B). To determine whether the spontaneous mutants are cheaters who do not produce public good oxalate, we also monitored the external pH, oxalate, and ammonia in LB supplemented with casamino acids or glucose with both strains. The external pH of BGR1 and BGR80S in either casamino acids- or glucose-rich conditions was maintained at a light acidic to neutral level due to the amount of oxalate produced, even though ammonia was highly produced by both strains grown in LB supplemented with casamino acids (Figures 8C–E). To determine whether spontaneous mutants emerged in minimal medium, populations of BGR1 and BGR80S growing in M9 minimal medium supplemented with 0.4% glucose were monitored for 8 days. The mutant was observed in BGR80S 6 days after subculture and comprised ∼40% of the total population (Supplementary Figure 7).

## Discussion

The genetic diversity of *B*. *glumae* strains, which are widely distributed in various niches, has been analyzed in many studies (Francis et al., 2013; Lee et al., 2016; Hussain et al., 2020; Choi et al., 2021; Cui et al., 2021). The clinical isolate AU6208 was reported to be more virulent on rice than the rice-pathogenic strains BGR1 and LMG 2196; however, nucleotide sequences showed 99.4% identity between the human- and plant-pathogenic strains (Cui et al., 2021). There was no significant difference in pathogenicity between Korean *B*. *glumae* isolates, despite the genetic diversity revealed by Tnp-PCR genomic fingerprinting (Choi et al., 2021). Likewise, no differences in pathogenicity were observed among the *B*. *glumae* isolates in this study, even though there were significant differences in genome structure, such as chromosomal merging.

It is increasingly recognized that, in addition to single-nucleotide changes, structural variations such as large genomic rearrangements can contribute to the evolution of an organism (Yeaman, 2013; Periwal and Scaria, 2015). Chromosomal rearrangements can disrupt an existing gene and create a new gene or chimeric gene product through gene fusions (Periwal and Scaria, 2015). In bacterial genomes, chromosomal rearrangements can affect gene expression by altering gene copy numbers, by changing the distance of a gene from the origin of chromosome replication (Rebollo et al., 1988). In this study, isolates of BGR76S, BGR22S, BGR28S, and BGR86S were in the same phylogenetic cluster as BGR80S and BGR80S, but their genome structure was completely different (Figure 1 and Supplementary Figure 1). The appearance of spontaneous mutants in BGR80S and BGR81S under nutrient stress was not observed in others of the same cluster. The genomic structural variations provide deeper insight into the bacterial genome arrangement with much higher resolution (Periwal and Scaria, 2015).

Acquired kanamycin resistance can be transferred from donors to recipients by horizontal gene transfer (Leungtongkam et al., 2018). The *aphA* gene inserted into BGR57S, BGR59S, BGR68S, BGR73S, and BGR82S might have been introduced into *B*. *glumae* cells by horizontal gene transfer of plasmid such as pSCR001. pSCR001 carries Omegon-Km originating from pJFF350 (Fellay et al., 1987, 1989; Giddens et al., 2007).

Naturally occurring *Vibrio cholera* strains with a single chromosome (in the form of merged chromosomes) have also been reported (Xie et al., 2017; Yamamoto et al., 2018). In *V*. *cholerae* strains NSCV1 and NSCV2, fusion junctions of chromosomes 1 and 2 contained more prophages, IS elements, repeats, and rearrangements compared to the prototypical two-chromosome *V*. *cholerae* genomes (Xie et al., 2017), but with no insertion of a foreign DNA fragment (unlike BGR80S and BGR81S). Similar to the virulence of the *V*. *cholerae* strains NSCV1 and NSCV2 seen in patients (Xie et al., 2017), BGR80S and BGR81S were associated with panicle blight symptoms in rice, because they still possessed virulence and toxoflavin genes (Supplementary Figures 3, 4). Since two replication origins were identified on the fused chromosomes of BGR80S and BGR81S (Figure 4), similar to *V*. *cholerae* NSCV1 and NSCV2 (Xie et al., 2017), it is worth exploring whether they are involved in replication, as well as how the chromosomal merging is maintained and under what conditions it reverts to two separate chromosomes.

*B*. *glumae* utilizes the amino acids in LB medium as its carbon source, but most amino acids are depleted during the stationary phase (Goo et al., 2012; Marunga et al., 2021). Heterogeneity is typically observed in liquid medium batch cultures when nutrient depletion starts, and it increases when there is environmental stress, such as nutrient depletion or metabolic stress (Gasperotti et al., 2020). Appearance of heterogenetic variants is a putative adaptive response to adverse environmental conditions within an aging culture (Ryall et al., 2012). Although the occurrence of bacterial variants during batch cultures is common, the ideal mutations for survival are unknown. For *B*. *glumae* isolates, *qsmR* mutants are common for fitness benefits, and show varying occurrence frequencies depending on the genomic traits of the strain. However, the molecular mechanisms and associated genomic structures involved in inducing *qsmR* mutation are unknown. We previously reported occurrence of *qsmR* mutation under metabolic stress (Goo et al., 2017), however it is not known whether appearance of *qsmR* mutant is a common phenomenon in *B*. *glumae* under physiological stress. Once the mutant is isolated, it cannot survive due to the alkaline extracellular pH resulting from its inability to biosynthesize oxalate in a QS-dependent manner as observed previously, since *B*. *glumae* is not viable above pH 8.5 (Goo et al., 2012, 2017).

Another question is whether the *B*. *glumae qsmR* mutants are cheaters that do not produce oxalate to benefit the population. Social cheaters emerge in populations growing in media requiring QS-regulated public goods, which increases the cost of cooperation (Sandoz et al., 2007; Dandekar et al., 2012). Based on two lines of evidence, the *qsmR* mutants isolated in this study are likely not cheaters. First, upon addition of casamino acids to the batch culture, a more significant amount of ammonia was released than in the plain LB, which increased the demand for oxalate (Figure 8). However, the *qsmR* mutant did not appear with daily addition of casamino acids. Second, the *qsmR* mutant appeared in M9 minimal medium, which scarcely requires oxalate (Supplementary Figure 7). Therefore, the *qsmR* mutants isolated during batch culture might have undergone adaptation in a nutrient-depleted state.

Our study suggests that genome structure is correlated with adaptation to nutrient stress conditions and the emergence of QS-dependent gene regulation defects, which provide increased fitness benefits in *B*. *glumae*. During the post-stationary phase, the changing nutritional environment is more akin to what bacteria experience in natural environments, where cells spend most of their time under starvation and stress conditions. Our experiment provides insight into how genomic structural variations influence adaptive evolution, revealing evolutionary changes in an organism that make it compatible with its habitat.

## Data availability statement

The datasets presented in this study can be found in online repositories. The names of the repository/repositories and accession number(s) can be found below: https://www.ncbi.nlm.nih.gov/ (SAMN10390410–SAMN10390413; SAMN29352428–SAMN29352458).

## Author contributions

MK, EG, and IH designed the experiments and wrote the manuscript. MK and EG performed the experiments. MK, JL, JK, EG, and IH analyzed the data. JL and JK contributed to the reagents, materials, and analysis tools. All authors contributed to the article and approved the submitted version.

## Conflict of interest

The authors declare that the research was conducted in the absence of any commercial or financial relationships that could be construed as a potential conflict of interest.

## Publisher’s note

All claims expressed in this article are solely those of the authors and do not necessarily represent those of their affiliated organizations, or those of the publisher, the editors and the reviewers. Any product that may be evaluated in this article, or claim that may be made by its manufacturer, is not guaranteed or endorsed by the publisher.

## Footnotes

1
https://pb-falcon.readthedocs.io/en/latest/
