# Supplementary material for: Influence of genomic structural variations and nutritional conditions on the emergence of quorum sensing-dependent gene regulation defects in Burkholderia glumae
Source: Front Microbiol. 2022 Jul 15;13:950600. doi: 10.3389/fmicb.2022.950600 (PMC9335073; doi:10.3389/fmicb.2022.950600)
Supplement: Supplementary file 1 [file Table_1.docx]

Supplementary Material

**Figure S1**. Synteny analysis of *B. glumae* isolates. Genomes of each isolate are compared to BGR1. The upper part is the BGR1 genome; the lower part is the genomes of other *B. glumae* isolates. The blue lines between genomes of two different isolates show the regions corresponding to each other; the red lines show the areas where inversion occurred. The vertical green lines indicate the locations of SNPs compared to BGR1; the vertical purple lines indicate INDELs. The darker the color, the more SNPs or INDELs occurred.


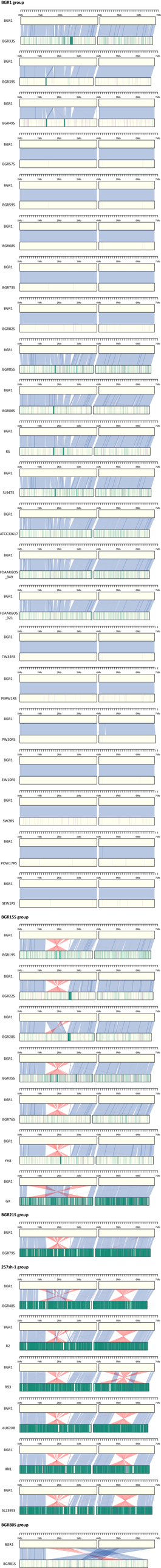


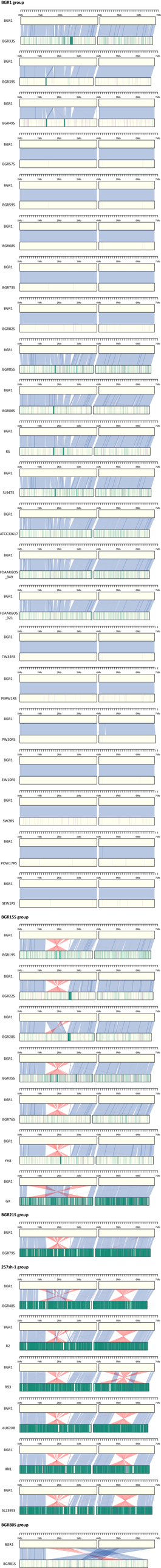


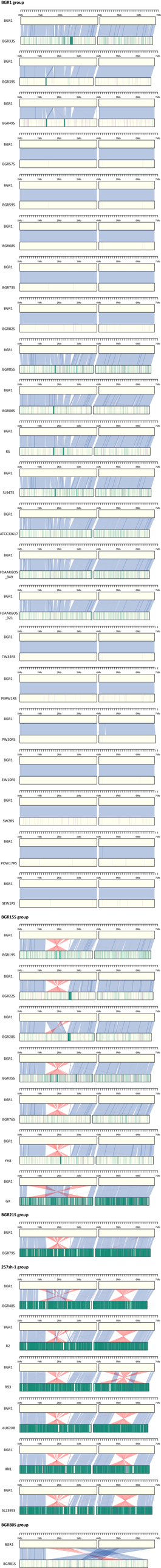


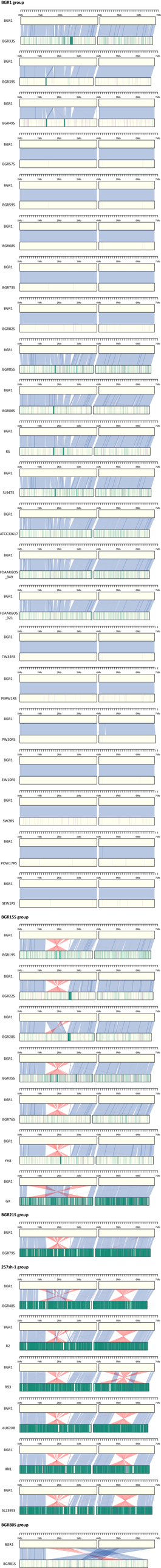


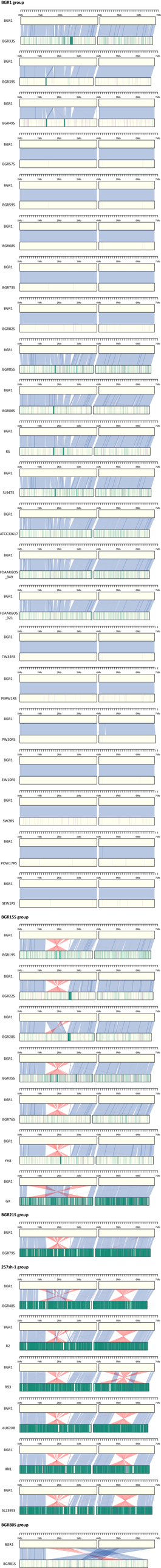


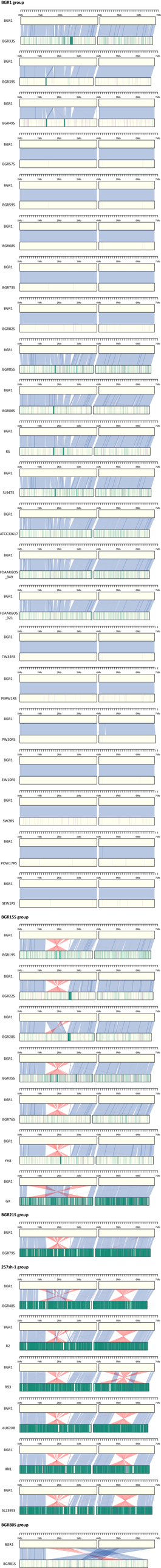


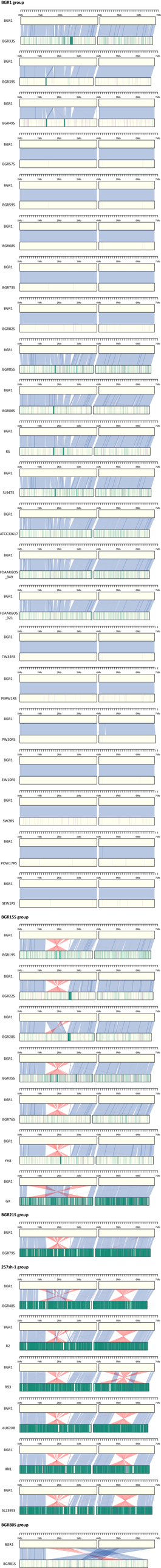


**Figure S2.** Kanamycin resistance of *B. glumae* isolates. It was observed whether the isolates could grow in LB medium containing kanamycin at a concentration of 50 mg/mL.


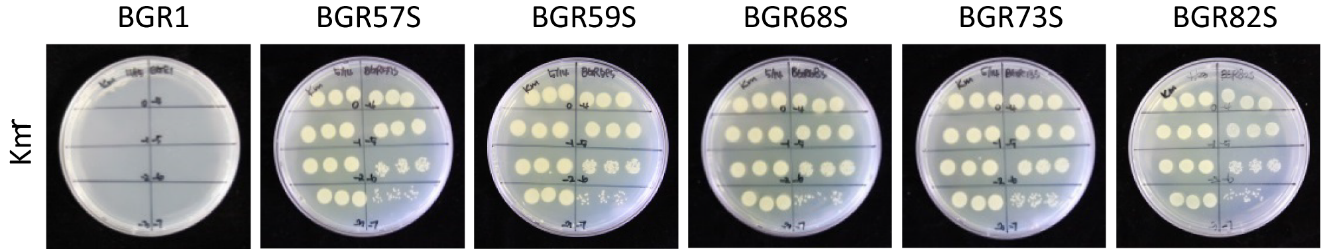


**Figure S3**. Disease symptoms of the indicated *B. glumae* strains in rice stems were photographed 7 days after inoculation. The numbers below the disease symptoms are the relative disease index scored by ImageJ software by comparison with the wild type values. Data represent the mean ± standard error (SE) of triplicates.


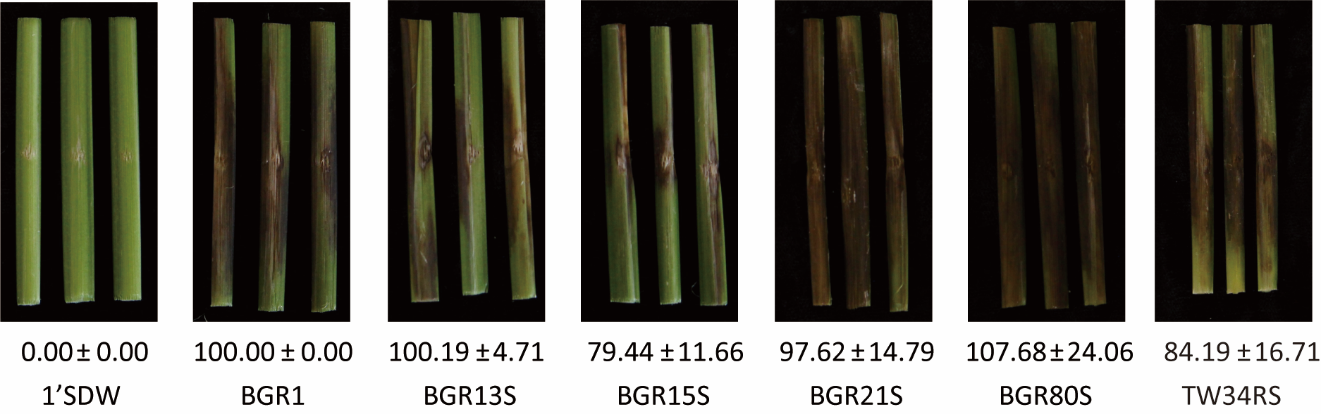


**Figure S4.** The *B. glumae* isolates produced (A) autoinducers and (B) toxoflavin to the same levels as those produced by the BGR1*.* C6-HSL; hexanoyl homoserine lactone, C8-HSL; octanoyl-HSL, T; toxoflavin.


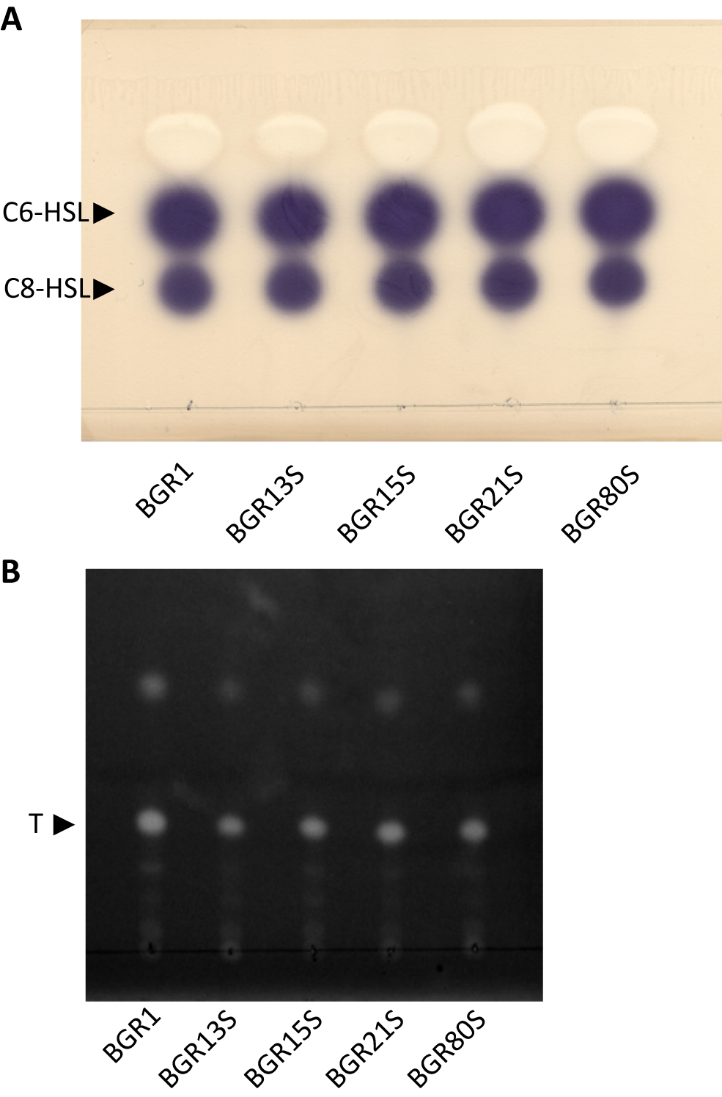


**Figure S5.** The pH changes of *B*. *glumae* isolates of BGR1, BGR13S, BGR15S, BGR21S, and BGR80S. The pH was measured at 1, 2, 4, 6, and 8 days after subculture. Data represent the mean ± SE of triplicates.


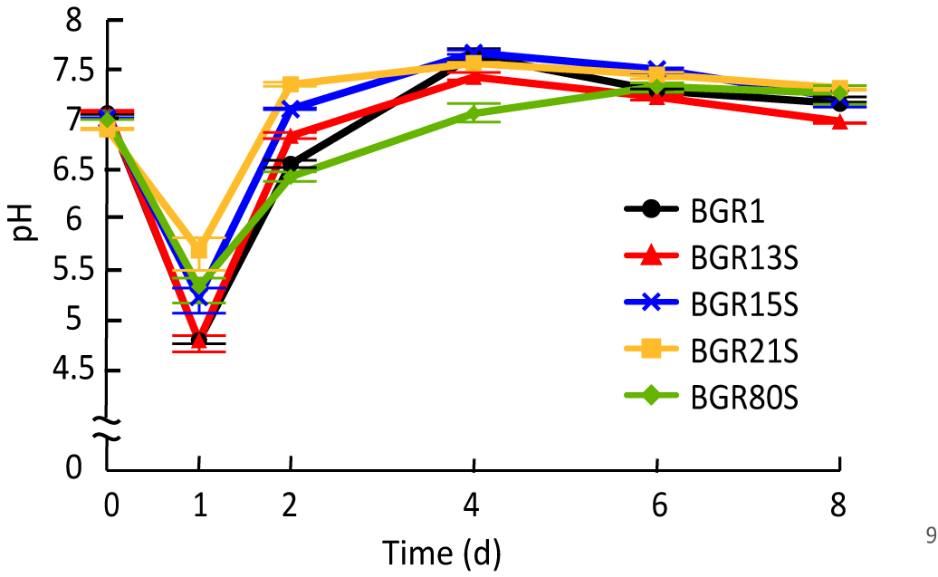


**Figure S6.** Cell viability and pH changes of the BGR1 and mutant. (A) Viable cell numbers and (B) pH were monitored at 6, 12, and 24 hours after subculture. Data represent the mean ± SE of triplicates.

**
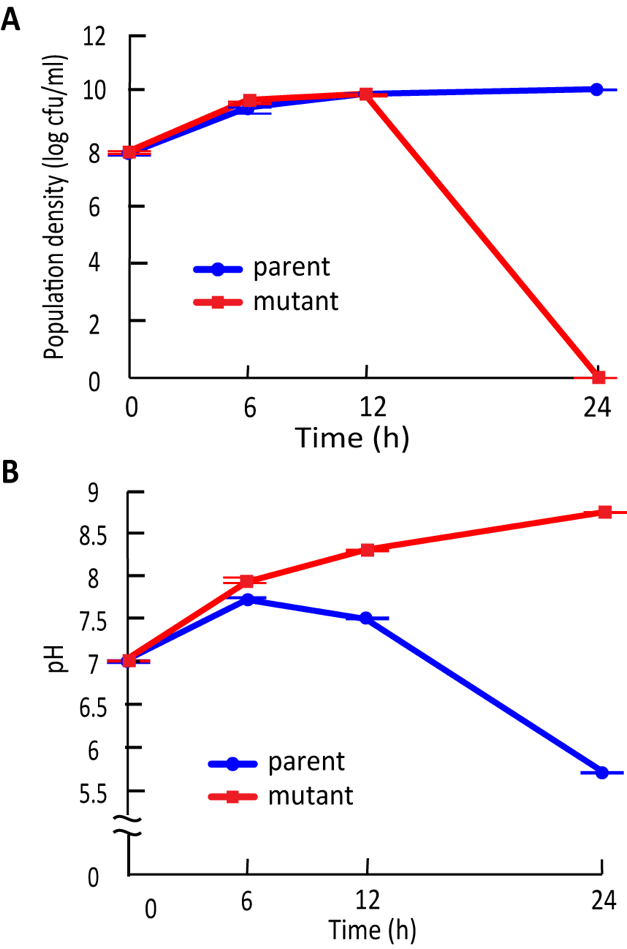
**

**Figure S7.** Appearance of spontaneous mutant in BGR80S during growth in M9 minimal medium supplementing 0.4% glucose. (A) Cell viability, (B) the occurrence rate of spontaneous mutant, (C) the external pH, (D) ammonia and (E) oxalate production of the BGR1 and BGR80S were monitored at 0, 2, 4, 6, and 8 days after subculture. Data represent the mean ± SE of triplicates.


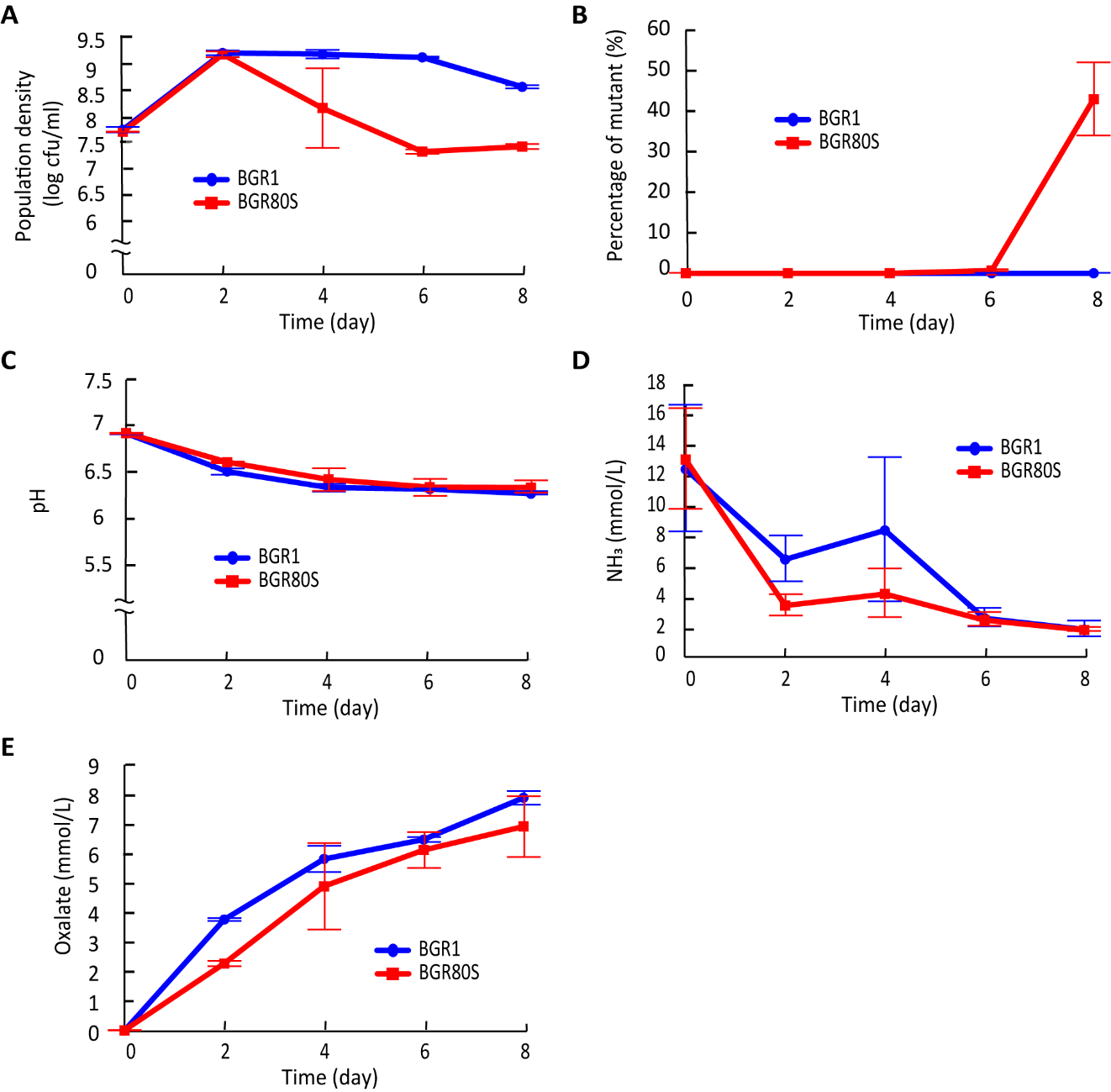


**Table S1.** List of primers used in this study

| Primers | Sequence (5' to 3') |
| --- | --- |
| tofI-F | TCAGGCCGCTTCGGGTTGCGACGCGCAA |
| tofR-B-R | TTTGGATCCGAATGACGCGCCCATGAG |
| qsmR-F | GGAAATGAGGGAGACCAGTCTGTCT |
| qsmR-R | CTTTCTTTCGACCGTTTACGGCGG |
| B-obcA-F | AGCTAGCGGATCCGGACGGATGGGGTCCGATTTTCGG |
| obcB-E-R | CATGCATGAATTCTCACCGCGTCACGCGTACCAGCT |

F, forward; R, reverse.
